# Critical Role of Somatosensation in Postural Control Following Spaceflight: Vestibularly Deficient Astronauts Are Not Able to Maintain Upright Stance During Compromised Somatosensation

**DOI:** 10.3389/fphys.2018.01680

**Published:** 2018-11-27

**Authors:** Recep A. Ozdemir, Rahul Goel, Millard F. Reschke, Scott J. Wood, William H. Paloski

**Affiliations:** ^1^Beth Israel Deaconess Medical Center, Harvard Medical School, Boston, MA, United States; ^2^Department of Health and Human Performance, University of Houston, Houston, TX, United States; ^3^Neurosciences Laboratory, Johnson Space Center, National Aeronautics and Space Administration, Houston, TX, United States; ^4^Human Research Program, Johnson Space Center, National Aeronautics and Space Administration, Houston, TX, United States

**Keywords:** sensory reweighting, postural control, spaceflight, somatosensory inputs, elderly

## Abstract

The free-fall of orbital spaceflight effectively removes the gravitational vector used as a primary spatial orientation reference on Earth. Sustained absence of this reference drives adaptive changes in the internal perception-action models of the central nervous system (CNS), most notably in the processing of the vestibular otolith inputs. Upon landing, the return of the gravitational signal triggers a re-adaptation that restores terrestrial performance; however, during this period, the individual suffers from a functional vestibular deficiency. Here we provide evidence of a transient increase of the weighting of somatosensory inputs in postural control while the CNS resolves these vestibular deficiencies. Postural control performance was measured before and after spaceflight in 11 Shuttle astronauts and 11 matched controls and nine elderly who did not experience spaceflight. A quiet-stance paradigm was used that eliminated vision, modulated the lower extremity somatosensory cues by subtly modulating the orientation of the support surface beneath feet of subjects in all groups. Additionally, in astronauts and matched controls, we challenged the vestibular system with dynamic head tilts. Postural stability on the landing day (R+0) was substantially decreased for trials with absent visual and altered somatosensory cues, especially those also requiring dynamic head tilts ( ± 5° @ 0.33 Hz) during which 20/22 trials ended prematurely with a fall. In contrast, none of the astronauts fell during eyes-closed, dynamic head tilt trials with unaltered somatosensory cues, and only 3/22 trials resulted in falls with eyes-closed and altered somatosensory cues, but static upright head orientation. Furthermore, postural control performance of astronauts was either statistically not different or worse than that of healthy elderly subjects during the most challenging vestibular conditions on R+0. Overall, our results demonstrate a transient reweighting of sensory cues associated with microgravity-induced vestibular deficiencies, with a significant increase in reliance on somatosensory cues, which can provide an effective reference even without vision and with dynamic vestibular challenges. The translation of these results to aging population suggests that elderly individuals with visual and vestibular deficits may benefit from therapeutic interventions enhancing sensorimotor-integration to improve balance and reduce the risk of falling.

## Introduction

All neurophysiological systems including sensorimotor networks controlling reflexive and coordinated voluntary motor behaviors have evolved to function in Earth’s gravity (Anken and Rahmann, 2002; Clement and Reschke, 2008). In the context of human upright stance and locomotion, gravitational sensory inputs from vestibular otolith organs serve as the primary sensory modality to establish a vertical spatial reference (Shumway-Cook and Woollacott, 2007; Macpherson and Horak, 2013; Pfeiffer et al., 2014) critical for controlling upright stance and terrestrial navigation. Adaptation of this essential spatial reference in response to sustained microgravity during spaceflight drives adaptive changes in the central nervous system (CNS) and leads to modification of internal models governing the input-integration-output characteristics of relevant sensorimotor repertoire (Clement and Reschke, 2008). The neural reorganizations that happen during spaceflight help to mitigate space motion sickness and optimize motor performance in the microgravity environment. However, these neural reorganizations are maladapted to gravitational constraints and, thus, significantly disrupt coordinated motor behaviors immediately upon returning to Earth (Paloski et al., 1992).

Upright stance control depends on the continuous integration of vestibular, visual, and somatosensory afference, and any ambiguous or disrupted inputs from one of these sensory modalities may cause destabilization of standing balance (Jacobs and Horak, 2007). Previous studies have consistently reported an increase in body sway and impaired upright stance control in astronauts following prolonged exposure to microgravity (Paloski et al., 1992, 1993; Wood et al., 2015). Misinterpretation of otolith signals (the otolith tilt-translation reinterpretation –OTTR-hypothesis), for example, has been proposed as possible mechanism of microgravity-induced maladaptive vestibular reorganization that degrade postural control and spatial orientation in astronauts while they are re-adapting to the return of gravitational inputs during early post-flight period (Young et al., 1984; Parker et al., 1985). One compensatory strategy the CNS is capable of employing during this maladapted early post-flight period can be the dynamic update of relevant internal models through sensory reweighting (Peterka, 2002). Sensory reweighting is an adaptive filtering process that regulates the relative contribution of each sensory modality to the internal model by down-weighting ambiguous afferences (e.g., vestibular) while up-weighting reliable sensory modalities to maximize overall gain and reduce signal-to-noise ratio (Polastri et al., 2012). For example, if the surface conditions are firm and stable, somatosensory inputs from the feet mechanoreceptors and ankle proprioceptors are more reliable than when standing on a soft and compliant surface.

Numerous previous studies have reported functional contribution (i.e., maintaining upright stance) of somatosensory inputs to postural control in healthy young adults with normal (Vuillerme et al., 2008) or disrupted vestibular function (Dietz et al., 1992; Mittelstaedt, 1992; Clapp and Wing, 1999; Kavounoudias et al., 2001; Bringoux et al., 2003; Modig et al., 2012), as well as in the elderly (Pasma et al., 2015) and clinical populations (Bronstein, 1999; Vaugoyeau et al., 2008). Research has shown increased reliance on somatosensory information under alcohol intoxication in healthy young adults (Modig et al., 2012), higher somatosensory weights in older adults with visual impairments (Pasma et al., 2015) and patients with unilateral vestibular loss (Peterka et al., 2011), and compensatory effects of using electro-tactile biofeedback during altered vestibular inputs in healthy young adults (Vuillerme et al., 2008). These studies have greatly improved our knowledge of the relative use of somatosensory information for maintaining postural control in various sensory contexts. However, the functional role of somatosensory inputs, signaling orientation of the body relative to surface-vertical, for stabilizing postural control during the early post-flight recovery period in vestibularly deficient astronauts is not well understood (Lowrey et al., 2014; Strzalkowski et al., 2015). Additionally, microgravity-induced musculoskeletal deconditioning and the transient vestibular deficiency can provide a unique model to better understand underlying mechanisms of impaired postural control in the elderly individuals with increased fall risks, and develop preventive rehabilitation protocols utilizing principles of sensory reweighing.

Thus, the primary goal of this study was to investigate the role of somatosensory inputs on postural control performance during disturbed/impaired vestibular conditions in vestibularly deficient astronaut subjects immediately after spaceflight. We administered dynamic head tilts during postural control tasks to further distort the accuracy of vestibular inputs following exposure to microgravity. Previous studies have shown that standard sensory organization tests (SOTs) may not be sensitive enough to detect subtle upright stance control dysfunctions in patients with vestibular disorders (Paloski et al., 2006; Mishra et al., 2009; Honaker et al., 2016), who may compensate upright stance control performance by task vigilance during SOTs. Therefore, modified SOTs with dynamic head movements have been suggested for better fall risk diagnosis during functional postural control performance assessments, both in elderly (Pang et al., 2011) and clinical populations (Mishra et al., 2009). Furthermore, when vision is absent, introducing an additional experimental challenge to the vestibular system by dynamic head movements would also allow us to better understand the compensatory role of sensory reweighting as a function of the availability of reliable somatosensory inputs. Therefore, we hypothesized that: availability of reliable somatosensory cues, in the absence of vision, will mitigate destabilizing effects of both impaired (due to microgravity) and distorted (due to head tilts) vestibular function during upright stance control. As a secondary purpose, we compared postural control performance from pre-flight and return day sessions’ of astronauts with healthy elderly individuals to better understand aging-related sensorimotor aspects of increased body sway.

## Materials and Methods

### Subjects

Postural control performance during disturbed vestibular function and compromised somatosensory inputs was systematically monitored in 11 astronauts (7 males, 4 females; age range 38–49 years) before and after short-duration (11–13 days) Shuttle flights, and 11 matched controls who followed the same timeline as astronauts but did not fly into space. Postural control performance of astronauts before and immediately after returning from spaceflight was also compared with nine healthy elderly subjects (3 males, 6 females; age range 73–86 years) to infer sensory mechanisms underlying postural control impairments in the elderly population. Each astronaut subject was a first-time flier. Each control subject was matched with an astronaut subject in terms of age (±4 years), sex, height (±5 cm), weight (±5 kg), and postural control performance [same quartile of Composite Equilibrium Score (EQ)]. Time spacing between pre- and post- “flight” sessions for controls was the same as that of matched astronauts. Postural control performance was assessed using computerized dynamic posturography (CDP). All subjects were participating in the CDP testing for the first time; hence any learning effects should have been similar for all groups. All astronauts passed NASA spaceflight physical examination prior to their missions, and control subjects had passed an Air Force Class III physical examination within 12 months of beginning the study. None of the astronaut or control subjects reported any history of balance or vestibular abnormalities. Elderly subjects were selected among those with no known neurological, cardiovascular, vestibular or musculoskeletal disorders, and no history of falls for at least 6 months before the start of the study. Overall health status of elderly subjects was screened by using the Physical Activity Readiness Questionnaire PAR-Q (Canadian Society for Exercise Physiology, 2002). Experimental protocols and voluntary participation procedures were explained to all subjects before they gave their written consent. All subjects were consented before inclusion. The selection criteria and experimental procedures for astronaut and control subjects were approved by the NASA Johnson Space Center (JSC) Committee for Protection of Human Subjects. The study protocol for elderly subjects was approved by the Institutional Review Board of the University of Houston.

**FIGURE 1:**
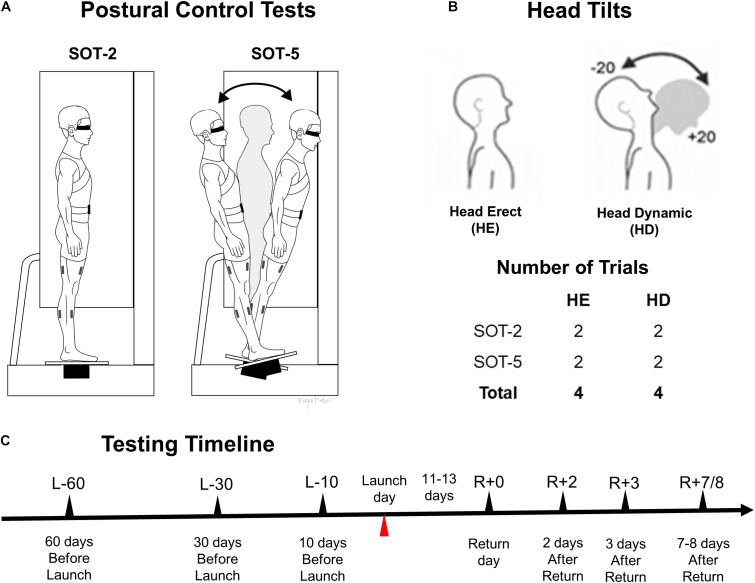
A schematic representation of study protocols and experimental conditions. Postural control tests (stable support surface, SOT-2 and sway-referenced support surface, SOT-5) are shown in **(A)**, while head conditions and number of trials for each postural control test are shown in **(B)**. **(C)** Shows a timeline of each session. All trials were conducted with eyes closed.

To establish pre-flight postural stability baseline data, each astronaut subject participated in four pre-flight testing sessions at JSC, occurring 141 (± 35), 133 (± 35), 50 (± 7), and 14 (± 1) days before launch (mean ± standard error of mean (SEM)), designated as familiarization (FAM), L-60, L-30, and L-10 sessions, respectively (Figure 1). The first pre-flight session, which occurred at least two days before the second pre-flight session, was considered FAM training for the postural tests using the standard SOTs, and data from this session were excluded from the analyses. The other three pre-flight sessions were considered to be independent estimates of a putatively stable individual. While nominally scheduled for 60, 30, and 10 days before the flight, launch schedules often shifted after one or more sessions had been completed. Since we did not have any reason to believe that the usual performance of astronauts would have been affected by launch delays, we accepted the actual timing while still classifying them based on expected timing. The first post-flight session (R+0) was performed at the Kennedy Space Center (KSC), Florida within 2–5 h after return from spaceflight using an experimental setup identical to that at JSC. All subsequent post-flight sessions were performed at JSC at 2 (R+2), 3 (R+3) and 7 or 8 (R+7/8) days after return from spaceflight. The second (L-60) and third (L-30) pre-flight sessions, and the third (R+3) post-flight session included the experimental postural tasks before and after exposure to short-radius centrifugation. However, only pre-centrifugation data are presented here. Astronaut and control subjects were instructed to avoid exposure to other unusual motion environments, strenuous physical activities or other experiments that might disrupt their recovery of balance function. Elderly subjects participated in a single session which included both familiarization and testing trials.

### Experimental Procedures and Data Collection

Postural control performance was evaluated using a modified CDP system (Neurocom Balance Manager, Natus Medical Incorporated, Pleasanton, CA, United States). The CDP system enabled SOT procedures, documented elsewhere (Paloski et al., 1992). During the FAM session, three trials of each of the six SOT conditions were carried out to familiarize astronaut and control subjects with the CDP system and to assess their baseline postural control performance which was also one of the metrics used to match astronaut and control subjects. During each experimental session (L-60, L-30, L-10, R+0, R+2, R+3, R+7/8), twelve 20 s trials were conducted with eyes closed using a combination of modulation of the sway of the support surface and head tilt conditions. The support surface was either fixed (SOT-2) or sway-referenced in the sagittal plane (SOT-5) in direct proportion to the estimated instantaneous center-of-mass (COM) sway angle (i.e., a gain of 1 was used). Subjects were instructed to either maintain the position of their head static and upright (head erect, HE) or perform continuous ± 20° dynamic (head dynamic, HD) pitch tilt oscillations at 0.33 Hz paced by an audible tone transmitted through lightweight headphones. Two trials were conducted for each of the HE and HD head conditions during both SOT-2 and SOT-5 support-surface conditions. Schematic representations of the experimental setup and postural task conditions used for astronaut and control subjects are shown in Figure 1. The trial order was counterbalanced across astronaut and control subjects and held constant across sessions. Throughout each trial, the astronaut or control subject was instructed to maintain a stable upright posture with arms folded across the chest. White noise supplied through headphones masked external auditory orientation cues. Infrared markers placed on the headset frame were used to quantify head position using an OptoTrak System (Model 3020, Northern Digital Inc., ON, Canada). Before beginning each dynamic head tilt trial, the test operator used real-time head position display information to guide the subject in achieving the desired head tilts, providing corrective instruction. Amplitude and phase of head pitch position during dynamic head tilts were obtained from both sinusoidal curve fits and detection of the maximum and minimum position in each cycle. Phase shift was made relative to the sinusoidally varying audio tone. Every dynamic head tilt trial began only after the operator has ascertained that the head tilts are approximately ± 20°. Once the dynamic head tilt trial began, the audible tone continued, but no feedback was provided for the remainder of the trial.

For specific details of the experimental procedures used with elderly subjects, please see Ozdemir et al. (2018). In brief, elderly subjects performed postural tasks with no head tilts under three different sensory conditions: (1) stable surface with eyes-open (SOT-1), (2) stable surface with eyes-closed (SOT-2), and (3) sway-referenced surface with eyes-closed (SOT-5). Two 90 s long trials were performed with 30 s testing duration for each of the three conditions continuously, without having a break among sensory conditions within each trial. Only the first 20 s of the data from the SOT-2 and SOT-5 conditions of the first trial were analyzed and presented here to compare with the performance of the astronaut subjects in their first session (L-60) and on return day (R+0).

### Data Reduction and Analyses

**FIGURE 2:**
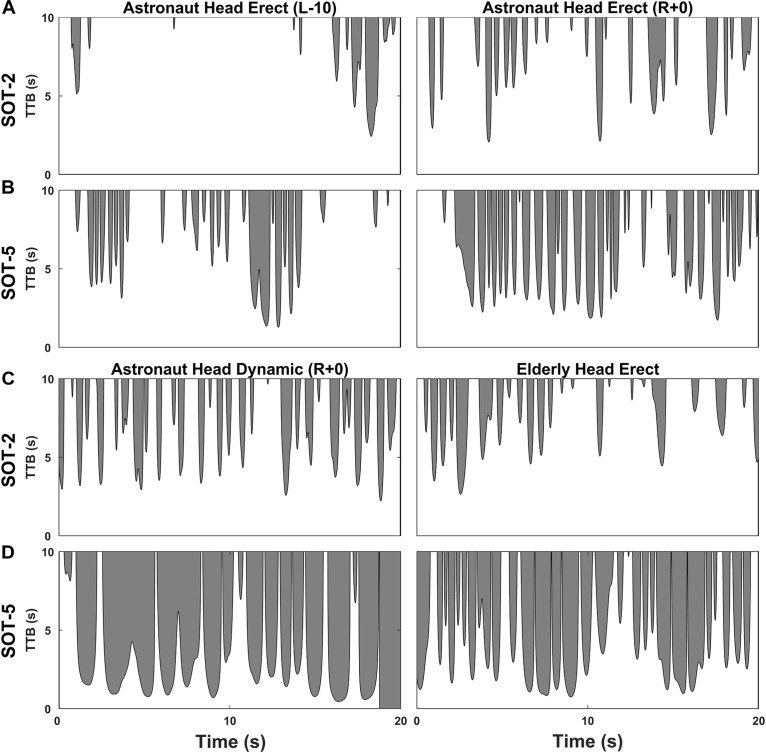
Representative time to boundary (TTB) time series for an astronaut and an elderly subject. Effects of spaceflight on postural control performance can be observed from head erect (HE) trials obtained from a representative astronaut subject before spaceflight (L-10) and immediately after landing (R+0) for both SOT-2 **(A)** and SOT-5 **(B)** conditions. Similarities in postural control performance between spaceflight and aging can be observed from head dynamic (HD) trials obtained on return day (R+0) in a representative astronaut subject and head erect (HE) trials obtained in a representative elderly subject for both SOT-2 **(C)** and SOT-5 **(D)** conditions. Black lines with shaded regions depict the TTB trace in anterior-posterior direction over each trial truncated at 10 s. Shaded areas indicate the iTTB (absolute) for each trial. The iTTB (%) is computed by dividing the iTTB (absolute) by the total area shown in the plot. The shaded region in **(D)** for an astronaut subject also show the time instance when TTB value hit zero, indicating a fall.

For the experimental sessions, the subject’s center of pressure (COP) was computed directly from force transducers in the support platform sampled at 100 Hz. The extremes of the feet defined the base of support. The COM was estimated based on a 2nd order low-pass Butterworth filter (0.85 Hz cutoff) applied to the COP (Ozdemir et al., 2013). Time-to-boundary (TTB) was calculated by dividing the instantaneous anterior-posterior (AP) COM distance to the boundary of the base of support by the instantaneous COM velocity (Forth et al., 2007). The TTB at each instance suggest the time it would take to reach the boundary of the base of support if you were to continue to move in the same direction and at the same speed. A higher value of TTB implies more stability. This measure has the advantage of combining both the spatial and temporal aspects of sway by also evaluating the influence of velocity (Haddad et al., 2006), and is sensitive to changes in stability limits with aging and support surface compliance (Haibach et al., 2007). The primary postural control performance measure was the integrated area of TTB (iTTB) below an arbitrary 10 s threshold that represents an estimate of relative stability over the entire trial (Figure 2). The iTTB is expressed as a fraction of the total area beneath the threshold (i.e., 10 s × trial duration) and is not affected by “falls” (subject raising a foot or arm to maintain balance), which are discrete events that cannot be considered part of the continuous EQ distribution. A lower value of iTTB represent higher stability.

The primary goal of this study was to investigate the role of somatosensory inputs on postural control performance in vestibularly deficient astronaut subjects immediately after spaceflight. SOT-5 trials for which the peak support surface tilt was less than 1° were discarded both in astronaut and in control subjects, as we do not expect any distortion of somatosensory cues in those trials. We chose to use 1° to discard trials as Peterka (2002) observed reweighting only when support surface perturbations were greater or equal to 2°. Such trials were identified by plotting peak support surface sway as a function of peak AP COM sway for SOT-5 trials across all head conditions for data from astronaut and control subjects. All data analyses were performed using customized MATLAB (MathWorks, Natick, MA, United States) scripts and functions.

Owing to sensory reweighting, we hypothesized that the availability of reliable somatosensory inputs, a stable platform in SOT-2, would reduce the destabilizing effects of HD tilts on postural control performance. To test this hypothesis, we calculated a series of sensory ratios using control and astronaut data from the R+0 day session. First of all, to follow similar interpretation of sensory ratios as used by ratios obtained using EQ (Neurocom, 2009), we subtracted iTTB (%) from 100 for these analyses, such that higher (100 – iTTB) % represents good balance. Somatosensory index (SI) was then calculated as a ratio of (100 – iTTB) % of HD by HE conditions and represents the subject’s ability to use input from the somatosensory system to maintain balance. Somatosensory reweighing index (SRwI) was calculated as a ratio of SI of SOT-2 by SOT-5. The SRwI measure helped us to test our primary hypothesis as it represents how well the availability of somatosensory inputs can compensate for destabilizing effects of HD. Vestibular index (VI) was calculated as a ratio of (100 – iTTB) % of SOT-5 by SOT-2 conditions and represents the subject’s ability to use input from the vestibular system to maintain balance. We also calculated somatosensory change index (SCI) as a ratio of SI of astronaut by control representing how much SI changed due to spaceflight, and vestibular change index (VCI) as a ratio of VI of astronaut by control representing how much VI changed due to spaceflight. SCI was calculated only for SOT-2 and not SOT-5 as during SOT-2, subjects are supposed to rely more on somatosensory inputs. Similarly, VCI was calculated only for HD and not HE as HD has been shown to be a more sensitive test to assess vestibular changes (Jain et al., 2010).

### Statistical Analyses

Preliminary analyses revealed that there was no statistical difference between iTTB values of first and second trial and thus data from only the first trial of each postural testing condition (SOT-2 and SOT-5; HE and HD as applicable) were used in statistical analyses for all groups (Control, Astronaut, Elderly). For all the “within-subject” comparisons (learning effect in control and astronaut subjects, and effects of spaceflight in astronauts), we used pairwise comparisons. If the underlying data were normally distributed [assessed using Shapiro–Wilk test (*p* > 0.05)], paired *t*-tests were carried out, else Wilcoxon Signed-rank tests were carried out. Independent sample *t*-tests (if data were normally distributed), or Mann–Whitney *U*-tests were performed for all the “between-subject” comparisons (control vs. astronaut pre-flight, control vs. astronaut return day, elderly vs. astronaut pre-flight, elderly vs. astronaut return day). Statistical significance was accepted at *p* < 0.05 (SPSS version 21, SPSS Inc., Chicago, IL, United States).

## Results

Figure 2 illustrates the time series data during different postural control performance conditions from a representative astronaut subject and an elderly subject.

### Learning Effects in Control Subjects

**FIGURE 3:**
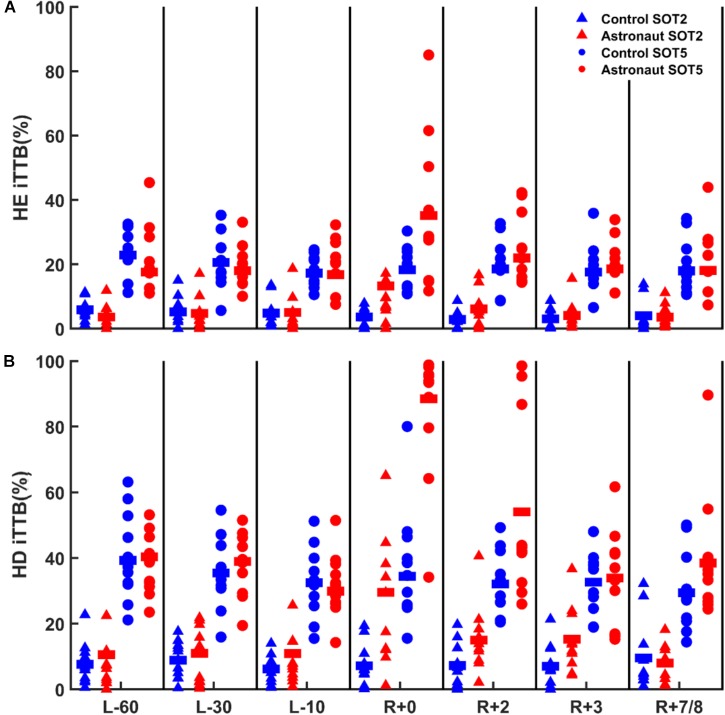
Postural control performance (iTTB) data from all control (blue symbols) and astronaut subjects (red symbols) for static (SOT-2, triangles) and dynamic (SOT-5, circles) support surface conditions during head erect (HE; **A**) and head dynamic (HD; **B**) head orientation conditions. Group means in each condition and testing session are shown by solid horizontal lines.

For the SOT-2 HE condition (Figure 3. blue triangles), there was a significant improvement (i.e., lower iTTB) at R+0 in comparison to L-60 (*Z* = 2.134, *p* = 0.033). However, no change was observed from R+0 to R+7/8 sessions (*p* > 0.05), indicating that postural control was fine-tuned from L-60 to R+0 sessions and was stabilized after R+0. For the SOT-2 HD condition, postural control performance was stable across sessions. During SOT-5, significant learning effect was observed between L-60 and L-10 in the HE condition [*t*_(8)_ = 3.161, *p* = 0.013], and then the performance remained stable for the following sessions. For the HD condition during SOT-5, there was a decreasing trend. However it was not significant (*p* > 0.05), and thus we can conclude that the postural control performance was stable across sessions.

### Postural Control Performance Before and After Spaceflight

Results of the independent sample *t*-tests showed no significant (*p* > 0.05) difference in postural control performance between control and astronaut subjects during any of the pre-flight sessions (L-60, L-30, and L-10) for either head condition (HE, HD) or support surface condition (SOT-2, SOT-5). Nor were any learning effects observed for astronaut subjects during pre-flight sessions for either head condition or support surface condition (*p* > 0.05).

To understand the effects of spaceflight on postural control, a series of paired comparisons were carried out between post-flight and pre-flight sessions’ in astronaut subjects. Since no learning effect was observed in astronaut subjects pre-flight, data of L-60 was used to compare with the data of the post-flight sessions of astronauts. For SOT-2, postural control performance was significantly reduced, i.e., higher iTTB, in the R+0 session when compared to the pre-flight session [R+0 vs. L-60 – HE: *t*_(10)_ = 3.020, *p* = 0.013; HD: *t*_(9)_ = 3.763, *p* = 0.004] for both head conditions (Figure 3, red triangles). For the HE condition in SOT-2, postural control performance became similar to the pre-flight level at the R+2 (R+2 vs. L-60: *p* > 0.05) session and remained stable during the following two post-flight sessions (*p* > 0.05 for R+2 vs. R+3, and R+3 vs. R+7/8). For the HD head condition in SOT-2, postural control performance returned to the pre-flight level only at the R+7/8 [R+2 vs. L-60: *t*_(9)_ = 3.540, *p* = 0.006; R+3 vs. L-60: *t*_(10)_ = 3.370, *p* = 0.007; R+7/8 vs. L-60: *p* > 0.05] session indicating a slower recovery in postural control performance during HD trials.

For SOT-5 trials, postural control performance significantly deteriorated in R+0 session when compared to the pre-flight session [R+0 vs. L-60 – HE: *Z* = 2.073, *p* = 0.038; HD: *t*_(9)_ = 6.539, *p* < 0.001] for both head conditions (Figure 3, red circles). Pairwise comparisons showed that in the HE condition, postural control performance returned to the pre-flight level at the R+2 session (R+2 vs. L-60: *p* > 0.05) and remained stable during the following sessions (*p* > 0.05 for R+2 vs. R+3, and R+3 vs. R+7/8). In the HD head condition, however, postural control performance was still impaired at the R+2 session compared to the pre-flight sessions (R+2 vs. L-60: *Z* = 2.803, *p* = 0.005) and returned to the pre-flight level only at the R+3 session and remained stable after that (*p* > 0.05 for R+3 vs. L-60, and R+3 vs. R+7/8), indicating longer recovery time when vestibular system was challenged.

### The Role of Somatosensory Inputs

For both astronaut and control subjects, we used data from the R+0 session to examine whether the availability of reliable somatosensory information could compensate for dynamic head tilt related performance decrements in balance control especially when the vestibular system is in a maladapted state due to microgravity effects on vestibular functioning.

**FIGURE 4:**
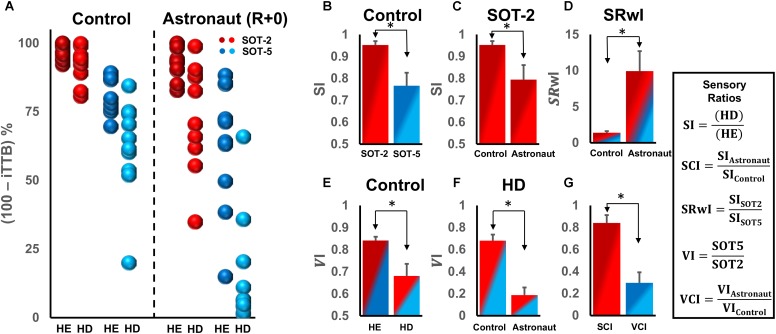
**(A)** Comparison of postural control performance as (100 – iTTB) % in controls and astronauts on the return day (R+0) for the two head and the two support surface tilt conditions. **(B)** Somatosensory index (SI) for the two support surface conditions for control subjects. **(C)** SI for control and astronaut subjects in SOT-2 condition on R+0. **(D)** Somatosensory reweighing (SRwI) index for control and astronaut subjects. **(E)** Vestibular index (VI) for the two types of head conditions for control subjects. **(F)** VI for control and astronaut subjects in the HD condition. **(G)** The somatosensory change index (SCI) for SOT-2 and the vestibular change index (VCI) for HD. The color shading in bars **(B–G)** is based on colors used to represent raw data in **(A)**. ^∗^*p* < 0.05.

First of all, we compared SI between the SOT-2 and SOT-5 conditions in control subjects (Figure 4B) and found that the SI for SOT-2 was significantly higher than the SI for SOT-5 (*Z* = 2.756, *p* = 0.006). This confirms, as expected, the importance of a stable, veridical, Earth-fixed reference for somatosensory inputs in HD compared to HE. Next, we compared the effects of spaceflight on the SI for SOT-2 (Figure 4C) and found that the SI was significantly lower in astronauts on the return day than in controls [*t*_(19)_ = 2.404, *p* = 0.027]. While this might suggest a reduction in reliance on somatosensory cues, it seems more likely that there could be some inaccuracies in somatosensory processing associated with spaceflight or that the alterations in the vestibular system associated with spaceflight were too profound to be fully compensated for by the somatosensory system. Furthermore, we compared the effects of spaceflight on SRwI (Figure 4D) and found SRwI to be nearly an order of magnitude higher in astronaut subjects on the return day than in control subjects (*Z* = 2.746, *p* = 0.005). This suggests a much higher reliance on somatosensory cues after spaceflight, even when they are inaccurate, confirming our primary hypothesis.

We then compared VI in control subjects between the two head conditions (Figure 4E) and found that the VI of HE was significantly higher than that for HD (*Z* = 2.756, *p* = 0.006). This confirms that, as expected, reliance on vestibular input decreases with HD. As expected, VI during HD for astronaut subjects on the return day was significantly lower than that in controls (Figure 4F, *Z* = 3.380, *p* < 0.001), clearly demonstrating a decreased reliance on vestibular inputs early after spaceflight.

Finally, we assessed relative decrements in performance after spaceflight associated with the vestibular and somatosensory systems by comparing SCI and VCI on the return day (Figure 4G). We found that the VCI was significantly lower than the SCI (*Z* = 2.803, *p* = 0.005), suggesting that the relative decrement in reliance on vestibular inputs was far greater than that for somatosensory inputs, resulting in a relative increase in reliance on somatosensory inputs.

Another functional performance metric is the number of fall (loss-of-balance) incidences observed under each test condition (Table 1). None of the subjects lost balance on any trial of SOT-2. The only two fall incidences observed in control subjects occurred during SOT-5 trials with HD (Table 1; bottom row). Conversely, on return day, all 11 astronaut subjects fell on at least one of two HD trials during SOT-5, and three astronaut subjects fell on one of the two HE trials during SOT-5. By R+2, recovery was well underway, as the incidence of falls on SOT-5 trials with HD decreased to 5/22, and beyond that, recovery was essentially complete, with only one fall observed in each of the final two test sessions.

**Table 1:** Number of falls across the head (HE and HD) and support-surface (SOT-2 and SOT-5) postural test conditions in 11 astronaut subjects during R+0, R+2, R+3, and R+7/8 sessions and in 11 control subjects during the R+0 session.

Subjects	Test	HE	HD
Astronaut R+0	SOT-2	0/22	0/22
	SOT-5	3/22	20/22
Astronaut R+2	SOT-2	0/22	0/22
	SOT-5	0/22	5/22
Astronaut R+3	SOT-2	0/22	0/22
	SOT-5	0/22	1/22
Astronaut R+7/8	SOT-2	0/22	0/22
	SOT-5	0/22	1/22
Control	SOT-2	0/22	0/22
	SOT-5	0/22	2/22


### Astronaut vs. Elderly Comparisons

**FIGURE 5:**
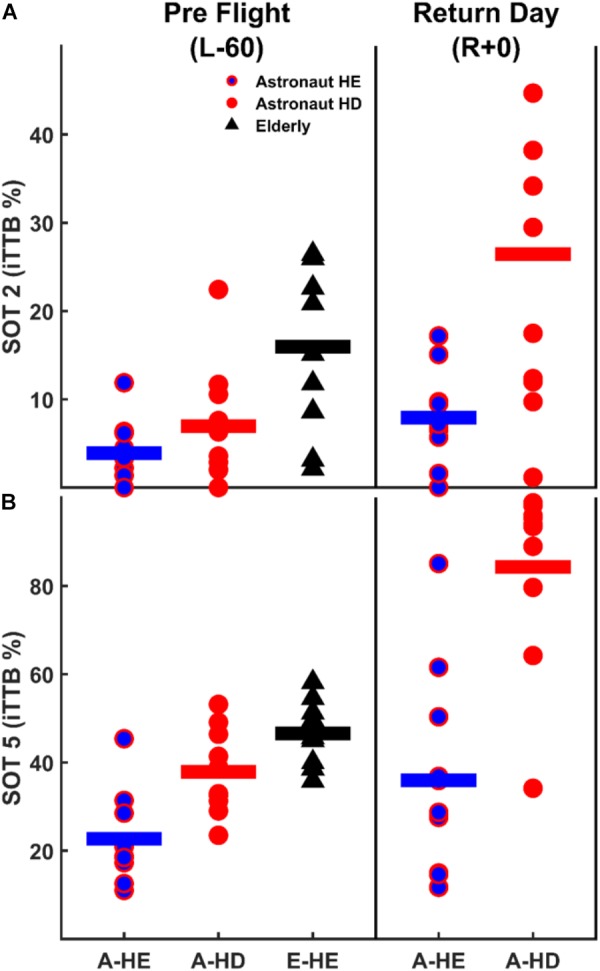
Postural control performance comparison between the astronaut and elderly subjects before (L-60) and immediately after (R+0) spaceflight both for SOT-2 **(A)** and SOT-5 **(B)** trials. Please note that the range of ordinate is different between panels.

To gain better insights regarding postural control impairments in the elderly subjects, we compared postural control performance of elderly subjects during SOT-2 and SOT-5 trials (only HE) with the astronaut subjects pre-flight (L-60) and immediately after return (R+0), in both head conditions (Figure 5). Pre-flight comparisons showed that postural sway was significantly higher in elderly subjects when compared to the HE [SOT-2: *t*_(18)_= -3.437, *p* = 0.007, SOT-5: *t*_(17)_= -5.810, *p* < 0.001], and the HD [SOT-2: *t*_(18)_= -2.347, *p* = 0.031, SOT-5: *t*_(18)_= -2.279, *p* = 0.035] conditions in astronaut subjects. The R+0 performance comparisons for SOT-2 trials showed no significant difference in performance between the HD condition in astronaut subjects and the HE condition in elderly subjects (*p* > 0.05), while in the HE condition in astronauts, postural sway was still significantly lower than that of elderly subjects in the HE condition [*t*_(18)_ = -2.370, *p* = 0.029]. For SOT-5 trials on R+0, however, astronaut performance in the HD condition was significantly worse [*t*_(17)_ = 5.190, *p* < 0.001] than that of elderly subjects in the HE condition, and no significant differences were found in HE trials between astronauts and elderly subjects (*p* > 0.05). Overall comparisons show that astronauts on R+0 (i.e., with a maladapted vestibular system) perform better than elderly subjects only when somatosensory cues are reliable. Our results also show that astronauts on the return day perform comparable to the elderly when vestibular inputs are disrupted through HD or when somatosensory cues are compromised, and perform worse than the elderly when vestibular inputs are disrupted through HD in compromised somatosensory condition.

## Discussion

The current study was designed to examine somatosensory contributions to upright stance control performance during distorted and maladapted vestibular functioning following short-term spaceflight in astronauts. Consistent with previous studies (Paloski et al., 1992; Jain et al., 2010), astronauts’ postural control performance was significantly degraded on the return day (R+0), in all postural control tasks and head conditions. Although somatosensory contributions to postural control may also have been degraded in astronauts, short-duration spaceflight primarily impaired vestibular functioning such that vestibularly deficient astronauts were able to maintain their upright stance when somatosensory cues were relatively reliable on a stable surface but inevitably fell when a sway-referenced surface further challenged the reliability of somatosensory cues in the absence of vision. Considering the incidence of falls during sway-referenced postural tasks, our analyses demonstrated the critical role of reliable somatosensory cues on functional upright postural control when the vestibular system is maladapted during the early post-flight period. Finally, comparable postural control performance between elderly and vestibularly deficient astronauts during challenging vestibular conditions on the return day supports current aging literature and suggests that therapeutic strategies enhancing sensorimotor integration can improve postural control performance in older adults.

### Spaceflight Disrupts Sensorimotor Control of Balance

Spaceflight causes distinct sensorimotor reorganizations due to the sustained absence of gravitational sensory inputs and lack of mechanical loading to the musculoskeletal system. Although adapted for a long-duration stay in space, these sensorimotor reorganizations are maladaptive to function in Earth’s gravity and, thus, pose serious postural control and locomotor challenges for astronauts by significantly increasing their risk of falling while they are restoring terrestrial performance during the early post-flight period. One critical aspect of monitoring post-flight sensorimotor re-adaptation is to employ valid testing paradigms to detect subtle changes in functional sensorimotor performance and determine the time course of sufficient recovery for astronauts such that they can safely return to their daily life activities. During the early history of human spaceflight, astronauts were allowed to return to their daily routines and duties two days after landing based on the results of standard clinical examinations (McCluskey et al., 2001; Clark, 2002). More recent reports (Jain et al., 2010; Wood et al., 2015) employing both standard and modified SOTs, and quantifying standing postural control performance with EQ scores showed substantially higher fall rates and longer recovery period during dynamic head movements with unstable support than standard SOTs with head erect and fixed-support. Our results, using iTTB as a postural control performance metric, further extend these previous reports that fall incidences and significantly higher body sway can still be observed in some astronauts when compared to their pre-flight levels, during dynamic head movements, on the last follow-up (R+7/8) examination. For example, during standard SOTs (HE) pre-flight postural control performance was found to be restored on R+2 both for SOT-2 and SOT-5. However, SOT trials with HD clearly showed that the pre-flight performance level was not achieved until R+3 for SOT-5, and R+7/8 for SOT-2, suggesting that fall risks are still present for certain astronauts up to a week following return from spaceflight during compromised visual and somatosensory conditions (e.g., walking in dim light or in dark, and over a compliant surface like sand). Due to our small sample size, however, prolonged recovery for HD during SOT-2 should be interpreted with caution since a closer examination of the data show higher variability in post-flight sessions, although no outliers were detected.

### Somatosensory Functioning Is Less Affected by Spaceflight and Critical During Maladapted Vestibular Functioning in Astronauts

Our analyses on the sensory ratios showed that the destabilizing effects of dynamic head movements on upright stance control change, as a function of reliable somatosensory inputs with respect to the surface-vertical, considerably in healthy controls but critically in vestibularly deficient astronauts. Specifically, when control subjects performed dynamic head movements blindfolded on a fixed support surface (SOT-2), providing reliable somatosensory inputs regarding body orientation, SI index was very high (0.95 ± 0.05) indicating that the performance difference between HE and HD is negligible in SOT-2 (Figure 4B red bar). However, when the dynamic head movements were performed on a sway-referenced support surface (SOT-5), compromising the reliability of somatosensory inputs, SI index was significantly lower (0.76 ± 0.06), suggesting a notably destabilizing effect of HD on upright stance performance even in healthy controls for SOT-5 trials (Figure 4B blue bar). Additionally, we also recorded two fall incidences during SOT-5 HD condition, and no fall during SOT-5 HE condition (Table 1), suggesting that the availability of reliable somatosensory cues may compensate for disrupted vestibular inputs in healthy controls.

However, this somatosensory driven compensation may become critical in vestibularly deficient astronauts immediately after spaceflight. Although we observed decreased SI and VI indices (Figures 4C,F) in astronauts, suggesting both impaired somatosensory and vestibular functioning, the degree of impairment was substantially higher in vestibular functioning following spaceflight. By comparing the ratio of changes in SI and VI between astronauts and healthy controls (Figure 4G), we showed a relatively high SCI but substantially decreased VCI, meaning astronauts were almost as good as healthy controls to utilize reliable somatosensory but were unable to use vestibular cues for compensating the destabilizing effects of HD. This suggests that somatosensory inputs are still relatively reliable sensory feedback source for vestibularly deficient astronauts, and thus they rely more on the less affected sensory system (somatosensory cues) to monitor their standing balance in the absence of vision while the CNS resolve transient vestibular deficiencies immediately upon return. We further supported these findings by showing an increased reliance into somatosensory weights in vestibularly deficient astronauts immediately after spaceflight (Figure 4D). In fact, availability of relatively reliable somatosensory cues was crucial for astronauts such that 20 out of 22 trials (% 90.9) resulted in falls when the validity of somatosensory cues for referencing gravitational vertical is further challenged during SOT-5. On the other hand, no single fall was observed when somatosensory cues could be used to infer gravitational vertical during SOT-2 trials. Thus the primary finding of this study is the critically functional role of somatosensory inputs from foot sole cutaneous receptors and ankle joint proprioceptors for maintaining upright stance in vestibularly deficient astronauts following spaceflight. Considering all the analyses we performed along with fall incidences, the difference between falling and standing for an astronaut during maladapted vestibular functioning seems to heavily depend on the reliability of somatosensory cues monitoring body sway with respect to the gravitational-vertical.

Promising hypotheses have been proposed such that the perceptual mechanism of vestibular (mal)adaptation following prolonged exposure to microgravity is explained mainly by reinterpretation of otolith inputs (Wood et al., 2015). Since otolith graviceptors only respond to translations in space, but not tilts, prolonged exposure to microgravity results in neglecting afferent signals from head tilts during spaceflight, and thus any head tilt is perceived as translation immediately upon returning to the Earth (Young et al., 1984; Parker et al., 1985). With an impaired vestibular function on the return day, performing dynamic head pitch movements on a fixed support surface poses further challenge to the postural control system by causing a unique ambiguity across sensory channels such that while vestibular inputs would be transmitting translation signals, somatosensory inputs from foot soles and ankle proprioceptors would be transmitting COP displacements and rotational torques of body sway, respectively. Owing to its plasticity, the CNS can resolve such sensory conflicts through sensory reweighting. Various form of this dynamic and compensatory sensory re-adaptation process has been increasingly investigated over the last two decades in postural control research (Peterka, 2002; Jeka et al., 2008; Vuillerme et al., 2008; Peterka et al., 2011; Modig et al., 2012; Polastri et al., 2012; Asslander and Peterka, 2014). In his seminal study, for example, Peterka (2002) monitored body sway characteristics on a sway-referenced platform with a progressive increase in surface sway angle. His results showed that blindfolded healthy subjects initially rely on the vertical surface reference to control body orientation for up to two degrees of surface sway angle but switches to a gravitational vertical reference by moving in the opposite direction to surface sway at larger sway amplitudes to maintain upright stance, indicating an accuracy based dynamic up-weighting and down-weighting of vestibular and somatosensory cues, respectively.

Although majority of studies have focused on sensory reweighting mechanisms of vestibular and visual inputs, recent studies consistently reported importance of somatosensory reweighting by evidencing increased reliance to mechanoreceptor inputs from the foot sole, ankle proprioceptor, and/or tactile cues to compensate for the destabilizing effects of different forms of disrupted or impaired vestibular and visual functioning on upright stance control (Vuillerme et al., 2008; Modig et al., 2012; Lowrey et al., 2014; Strzalkowski et al., 2015). Our results further extend these findings that reliable somatosensory cues from a firm and stable surface are important in healthy adults to stabilize upright stance during disrupted vestibular function (i.e., HD condition), but crucial in vestibularly deficient astronauts to prevent them from falling during the early period of post-flight recovery.

Although our data suggest a transient increase of the weighting of somatosensory inputs to upright stance control, caution should be taken to understand falls during dynamic head tilts in vestibularly deficient astronauts. It is obvious that, in addition to the absence of reliable somatosensory cues, many other sensory and musculoskeletal factors might have also contributed to the inevitable falls observed in astronauts during dynamic head movements on sway-referenced surface conditions. For example, as previously reported in patients with the bilateral vestibular loss (Peterka, 2002), vestibularly deficient astronauts might have failed to employ functional sensory reweighting during sway-referenced support surface condition (SOT-5) which optimally requires down-weighting of somatosensory and up-weighting of vestibular inputs. It is likely that astronauts’ increased reliance on somatosensory cues was maladapted during SOT-5 trials such that they were unable to switch from a surface reference to a gravity reference. Another important aspect to consider in falling astronauts is microgravity-induced musculoskeletal deconditioning: that can cause a decline in muscle stiffness and loss of force and power due to prolonged unloading; which may all contribute to falls (Wood et al., 2015). Considering all these mechanisms, however, we cannot rule out increased reliance on somatosensory cues when vestibular inputs are disrupted in astronauts during upright stance control task on the return day since we have observed the same strategy in control subjects with intact vestibular and musculoskeletal functioning. Our comparisons for the effects of dynamic head movements within the same sensory condition (Figure 4) strongly supports increased weighting of somatosensory inputs such that dynamic head movements do not further destabilize postural control performance on a fixed surface, but only on the sway-reference surface condition.

### Sensorimotor Impairments in the Elderly

In many ways, microgravity-induced physiological adaptations including overall deconditioning in musculoskeletal and cardiovascular systems, and a general decline in sensorimotor functions resemble the physiology of aging (Vernikos and Schneider, 2010). In this respect, spaceflight can be considered a unique model (Young, 1993) to probe underlying mechanisms of aging-related postural control impairments as older adults are long known to have increased body sway and thus highly prone to falls (Horak, 2006). Comparing postural control performance between elderly subjects and astronauts, our data suggest that the increased body sway in elderly subjects, in the absence of vision, can be attributed to vestibular deficiencies mainly, but also to somatosensory deficits. Post-flight comparisons indicate vestibular deficiencies in elderly subjects such that astronauts perform either comparable to or worse than elderly individuals on a sway referenced platform with HE and HD trials, respectively. No differences were also found between astronauts and elderly when astronauts perform HD trials on a stable support surface. Altogether post-flight comparisons suggest that astronauts perform a lot more comparable to elderly during the most challenging vestibular conditions (Figure 5 right panels). However, when somatosensory cues are reliable during the fixed support surface condition, better postural control performance in astronauts either during pre-flight comparisons with HD trials or post-flight comparisons with HE trials suggest somatosensory deficits or compromised reweighting of somatosensory cues in elderly subjects. Alternatively, a general decrease in musculoskeletal function with age should also be considered as an important factor for increased postural sway in the elderly during challenging sensory conditions. Overall, our findings are in agreement with many previous reports arguing that the vestibular system degenerates the most with age (Horak et al., 1989; Sturnieks et al., 2008; Barin and Dodson, 2011; Faraldo-Garcia et al., 2012; Liston et al., 2014b), and sensory re-weighting is slower and/or compromised during challenging upright stance conditions in healthy elderly adults when compared to young adults (Pavlou et al., 2004; Jeka et al., 2010).

Many implications can be derived from this study. A firm, stable surface for standing and walking can compensate for challenges associated with reduced vision (darkness, smoke-filled cabin) and dynamic head movement requirements immediately after landing, which can be very critical for safe egress. The translation of these results to aging population suggests that elderly individuals with visual and vestibular deficits may benefit from therapeutic interventions (Liston et al., 2014a) enhancing sensorimotor integration to improve postural control and reduce the risk of falling.

## Ethics Statement

This study was carried out in accordance with the recommendations of “The Protection of Human Research Subjects, NASA Flight IRB” with written informed consent from all subjects. All subjects gave written informed consent in accordance with the Declaration of Helsinki. The study protocol for astronaut and control participants was approved by NASA Flight IRB, and for elderly participants was approved by the IRB of the University of Houston.

## Author Contributions

RO helped with data analyses, interpretation of results, and writing of the manuscript. RG also helped with data analyses, interpretation of results, and writing of the manuscript. MR and SW helped with the study design and data collection. WP designed the study, and helped with data collection, interpretation of results, and revision of the manuscript.

## Conflict of Interest Statement

The authors declare that the research was conducted in the absence of any commercial or financial relationships that could be construed as a potential conflict of interest.

## Abbreviations

AP: anterior-posterior
CDP: computerized dynamic posturography
CNS: central nervous system
COM: center of mass
COP: center of pressure
EQ: equilibrium score
FAM: familiarization
HD: head dynamic
HE: head erect
iTTB: integrated time to boundary
JSC: Johnson Space Center
KSC: Kennedy Space Center
L-x: x days before launch of spaceflight
OTTR: otolith tilt-translation reinterpretation
PAR-Q: physical activity readiness questionnaire
R+x: x days after return from spaceflight
SOT: sensory organization tests
TTB: time to boundary
